# Strengthening research capacity through an intensive training program for biomedical investigators from low- and middle-income countries: the Vanderbilt Institute for Research Development and Ethics (VIRDE)

**DOI:** 10.1186/s12909-022-03162-8

**Published:** 2022-02-14

**Authors:** Holly M. Cassell, Elizabeth S. Rose, Troy D. Moon, Halima Bello-Manga, Muktar H. Aliyu, Wilbroad Mutale

**Affiliations:** 1 Vanderbilt Institute for Global Health, Vanderbilt University Medical Center, 2525 West End Avenue Suite 750, Nashville, TN 37203 USA; 2 Departments of Pediatrics, Health Policy, and Medicine, Vanderbilt University Medical Center, Nashville, TN USA; 3 Department of Hematology and Blood Transfusion, Barau Dikko Teaching Hospital/Kaduna State University, Kaduna, Nigeria; 4 Department of Health Policy and Management, School of Public Health, University of Zambia, Lusaka, Zambia

**Keywords:** Capacity strengthening, Research training, Global health, Low- and middle-income countries

## Abstract

**Background:**

Capacity strengthening initiatives aimed at increasing research knowledge and skills of investigators in low- and middle-income countries (LMICs) have been implemented over the last several decades. With increased capacity, local investigators will have greater leadership in defining research priorities and impact policy change to help improve health outcomes. Evaluations of models of capacity strengthening programs are often limited to short-term impact. Noting the limitations of traditional output-based evaluations, we utilized a broader framework to evaluate the long-term impact of the Vanderbilt Institute in Research Development and Ethics (VIRDE), a decade-old intensive grant development practicum specifically tailored for investigators from LMICs.

**Methods:**

To assess the impact of VIRDE on the research careers of alumni over the past 10 years, we surveyed alumni on research engagement, grant productivity, career trajectory, and knowledge gained in grant writing. Descriptive statistics, including means and total counts, and paired sample t-tests were used to analyze the data.

**Results:**

Forty-six of 58 alumni completed the survey. All respondents returned to their home countries and are currently engaged in research. Post-VIRDE grant writing knowledge ratings were significantly greater than pre-VIRDE. The number of respondents submitting grants post-VIRDE was 2.6 times higher than before the program. Eighty-three percent of respondents submitted a total of 147 grants post-VIRDE, of which 45.6% were awarded. Respondents acknowledged VIRDE’s positive impact on career growth and leadership, with 88% advancing in career stage.

**Conclusions:**

Gains in grant writing knowledge and grant productivity suggest that VIRDE scholars built skills and confidence in grant writing during the program. A substantial proportion of respondents have advanced in their careers and continue to work in academia in their country of origin. Results show a sustained impact on the research careers of VIRDE alumni. The broader framework for research capacity strengthening resulted in an expansive assessment of the VIRDE program and alumni, illuminating successful program elements and implications that can inform similar capacity strengthening programs.

## Background

Despite commitment by national governments and international funding, health outcomes in many low- and middle-income countries (LMICs) lag behind other nations [1]. Improving health outcomes may be linked to improved local academic biomedical research in these countries [2–7]. Effective, transformative research leaders can contribute to advances in responding to infectious and non-communicable diseases in their communities [8]. Over the past several decades, there has been increased emphasis on strengthening research knowledge and skills of investigators in LMICs [9]. With increased research skills and knowledge, local investigators could have greater leadership in defining priority research topics, as well as participating in data analysis and dissemination of results. Such leadership and influence would place investigators in prime positions to impact policy change to help improve health outcomes.

Many models of research capacity strengthening have been implemented over the years [10]. Programs have ranged from short-term, in-country workshops to multi-year, out-of-country degree programs and have focused on either a single topic area or multiple areas. Instruction is often conducted in-person, while some programs have utilized distance learning options [11, 12]. Beyond didactic lectures on research methodologies, common program elements include mentorship and participating in field placements or team-based projects [11, 13–20]. Programs are frequently implemented through multi-country partnerships, whether between high-income countries (HICs) and LMICs or a regional consortia of LMICs [7, 21–29]. Most programs report successful short-term impacts on increased knowledge among program participants, but some authors have criticized these capacity strengthening activities for not having a greater long-term impact on health outcomes [3, 9, 30]. Additional critiques of these programs include a lack of standard definitions, frameworks, and evaluation systems [3–5, 10, 31–33].

Reviews of capacity strengthening programs have noted difficulties in equipping scholars to work within structural challenges at institutional and country levels. Such challenges include a lack of funding, salary support, protected time to conduct research, research personnel and infrastructure, and mentorship [34–43]. Without adequate financial support, graduates are unable to support their research endeavors and may supplement their salary through consultancies or clinical engagements, thus decreasing the amount of time they can dedicate to the development and implementation of research. A lack of research administrative support as well as other research infrastructure, such as laboratory equipment and internet, can hinder research productivity. A lack of support from institutional leadership and mentors in the field can also dissuade enthusiastic researchers from pursuing their work. Further, due to differences in research environments and resources between LMICs and HICs, outcome measures commonly used in HICs, including publications in peer-reviewed journals, conference presentations, and funded grant applications, may not reflect accurately the research capacity and productivity in LMICs.

Noting the challenges that LMIC researchers encounter in sustaining a research career and inadequacies of traditional productivity metrics, Cooke created a framework to guide and evaluate research capacity strengthening programs [44]. The framework was built upon six principles that include building skills and confidence; remaining close to scholars’ practice; linking with networks and growing collaborations; improving health outcomes through dissemination of research findings; providing structures for sustainability and continuity; and supporting scholars’ continued research. Each of these principles operate at individual, team, organization, and supra-organizational levels.

In 2011, the Vanderbilt Institute in Research Development and Ethics (VIRDE) program was launched to support junior investigators in LMICs. VIRDE utilized Cooke’s six principles at the individual level to build a unique program that trains investigators in grant writing and research ethics, with an aim for program scholars to attain funding support, sustain their research career, and become research leaders who positively impact health outcomes through policy change. The four-week, intensive, mentored practicum takes place in-person at Vanderbilt University in Nashville, Tennessee, USA. VIRDE includes state-of-the-art didactic sessions promoting competencies in grant writing and the responsible conduct of research. These sessions are coupled with dedicated time for hands-on, mentored practical skills building in grant writing, culminating in a grant application that is ready for submission for external funding. Scholars also engage in laboratory rotations, graduate-level courses, and regular meetings with a personalized mentoring committee.

Evidence-based quality improvement is an essential element of the VIRDE program. Annual program evaluations inform program modifications and enhancements for the next iteration. Additional bi-annual check-ins with program alumni have contributed to quality improvement efforts and served to maintain networks and support alumni. To assess long-term impact, a program evaluation questionnaire utilizing Cooke’s principles was sent to all VIRDE alumni during the tenth year of the program. This paper illustrates the results of the program evaluation, assesses the impact of the program over the last decade, and describes lessons learned.

## Methods

### Participants

The VIRDE program was designed for biomedical and public health investigators in LMICs. Initially the program was developed to bolster research independence of junior faculty affiliated with Vanderbilt Institute for Global Health (VIGH) partner institutions in Africa, Asia, and Latin America, but has since expanded to include scholars affiliated with additional institutions. Partner institutions in LMICs manage the initial round of the selection process, identifying appropriate candidates. Selected applicants then complete an online application, including submission of their curriculum vitae or National Institutes of Health (NIH)-style biosketch, a draft proposal based on an NIH R21 grant format, and support letters from their in-country mentors. Prior to selection, applicants are required to give a proposal presentation online to the VIRDE Selection Committee. Priority is given to applicants who submit a compelling grant proposal idea and identify committed mentors from their home institution and Vanderbilt. Scholarships for VIRDE participants are supported by institutional funds and VIGH collaborative US government and foundation-sponsored training and research grants with LMIC partner institutions.

### Curricular elements

Cooke’s six principles of research capacity strengthening were incorporated into the VIRDE curriculum through seminars, mentoring teams, and on-going post-program collaborations. The VIRDE curriculum includes over 40 contact hours of specially tailored seminars in grant writing, grant administration, research ethics, and career development taught in person or virtually by Vanderbilt faculty and senior staff, VIRDE alumni, and LMIC partner faculty. Examples of grant writing seminars include funding strategies, effective writing of standard grant sections, and administrative elements of grant management. Research ethics seminars cover topics related to the fundamentals of research ethics, ethical research design, international standards of clinical trials, and ethics of authorship and collaborative partnerships. Career development seminars focus on leadership and strategies for success as an academic researcher.

### Training program structure

During the program, scholars participate daily in several hours of didactic workshops led by VIRDE faculty. When not in workshops, scholars hone their grant proposal and engage in various capacity strengthening activities at Vanderbilt, including attending courses and seminars, visiting laboratories and research cores, and meeting with potential collaborators. Scholars are supported by dedicated mentor teams from their home country and Vanderbilt-based mentors via meetings held several times each week during the course. With guidance from their mentors, scholars develop a grant proposal in stages by completing weekly writing assignments. The course concludes with a mock NIH-style grant review of the proposals by a committee comprised of Vanderbilt faculty, VIRDE alumni, VIRDE scholars, and invited experts. Scholars leave the program with a strong draft of a grant proposal and a submission plan. Upon returning to their home country, scholars continue to receive support from their mentoring team and administrative technical assistance from Vanderbilt staff.

### Evaluation and analysis

To assess the impact that the VIRDE program has had on the research careers of program alumni over the past 10 years, the authors developed an online program evaluation that was sent to all VIRDE alumni (*n* = 58). REDCap, a secure online platform for survey development and database management [45, 46], was used to design the survey instrument and email survey invitations and reminders. The instrument had 223 fields to query respondents on research engagement, grant productivity before and after VIRDE, career trajectory, leadership roles, collaborative activities, VIRDE program elements, and knowledge gained in grant writing and research ethics. Answer fields included checkboxes and open-ended responses. For questions related to grant writing and research ethics knowledge, respondents were asked to rate their knowledge prior to and after attending VIRDE on a five-point scale, ranging from strongly disagree (one) to strongly agree (five) for 10 elements of grant writing and a four-point scale, ranging from uninformed (one) to expert (four) for 18 elements of research ethics.

Descriptive statistics, including means and total counts, were used to analyze responses for the questions with checkboxes. Total counts were used to compare responses about grant awards before and after VIRDE. Where applicable, total counts were converted to a percentage of the total respondents for that question. Changes in knowledge scores were determined by calculating the mean response value for each question, both before and after VIRDE. Then, the pre-VIRDE mean was subtracted from the respective post-VIRDE mean for each question and the mean change was converted to a percentage. To analyze changes in knowledge before and after VIRDE, paired sample t-tests were conducted to determine statistical significance. Heat maps were created to visually highlight change in knowledge.

The authors have served as VIRDE instructors/mentors and the first author is also the program director and co-founder. To establish distance and maintain neutrality, data was analyzed by research assistants who were new to the program and survey responses were deidentified. Respondents provided their consent to participate in this evaluation and were assured of confidentiality in their responses. The study protocol and evaluation instrument were approved by the Vanderbilt University Institutional Review Board (#200133).

## Results

### Demographics

Fifty-eight LMIC academic biomedical investigators, including NIH-Fogarty International Center training grant alumni participated in VIRDE from 2011 to 2019, of which 34% were women. Forty-six alumni completed the survey. Two additional alumni began the survey but did not complete it, bringing the total responses for some questions to 47 or 48, depending on where the respondent stopped. With 48 out of 58 responses, the sample has a margin of error of 5% at the 90% confidence level. Of the survey respondents (*n* = 48), 68.8% were male and 31.3% were female (Table 1). A majority of participants held a terminal degree including an MD, MBBS, or equivalent (39.6%) or a PhD (37.5%). When stratifying the highest level of education achieved by sex, slight differences arose. More women than men (46.7% versus 36.4%, respectively) held an MD, MBBS, or equivalent degree as their highest degree, while more men than women (45.5% versus 20.0%, respectively) held a PhD.

**Table 1:** Demographic information of survey respondents (*n* = 48), Vanderbilt Institute for Research Development and Ethics (VIRDE)

Category	Total [Number (Percent)]
Respondents	
*Female*	15 (31.3%)
*Male*	33 (68.8%)
Highest degree attained	
*MD, MBBS, or equivalent*	19 (39.6%)
*PhD*	18 (37.5%)
*Master degree*	10 (20.8%)
*Bachelor degree*	1 (2.1%)
Country of residence	
*Brazil*	1 (2.1%)
*China*	1 (2.1%)
*Ghana*	6 (12.5%)
*Mozambique*	8 (16.7%)
*Nigeria*	10 (20.8%)
*Pakistan*	2 (4.2%)
*Tanzania*	3 (6.3%)
*Zambia*	15 (31.3%)
Employment sector (respondents could check multiple sectors)	
*Academia*	37
*Government*	10
*Non-government (domestic)*	4
*Non-government (international)*	3
Proportion of professional time dedicated to research	
*0–29%*	5 (10.4%)
*30–59%*	30 (62.5%)
*60–100%*	13 (27.1%)

Roughly 90% of respondents came from sub-Saharan Africa, with over 80% residing in one of four countries, Zambia (31.3%), Nigeria (20.8%), Mozambique (16.7%), or Ghana (12.5%). Other countries included Brazil, Bangladesh, China, Kenya, Pakistan, and Tanzania. All but one respondent has remained in their home country since completion of VIRDE.

Most respondents (77.1%) reported being employed in academia. Smaller percentages reported working in government institutions (20.8%) or non-governmental organizations (14.6%). Almost all respondents (92.0%) reported currently working at the same type of organization as they did during VIRDE, with many (88.0%) having advanced in their career stage since their participation in VIRDE. All respondents except one “*agreed”* or “*strongly agreed”* with the statement “*VIRDE contributed to my career advancement.”*

### Research engagement and grant writing

All respondents reported they currently work in some research capacity, although the hours devoted to research differed. The majority of respondents (62.5%) reported spending approximately 30 to 59% of their professional work time on research. Smaller proportions of respondents reported spending greater than 60% or less than 30% of their time on research (27.1 and 10.4% respectively).

Respondents were asked to select factors that enabled them to pursue research. The most common selected factor was “*a personal desire to do research”* (70.2%). Other factors selected by at least half of respondents included “*a positive research culture at their institution”* (64.6%) and “*mentorship”* (50%).

Respondents were requested to select factors that had the greatest impact on their ability to successfully develop research grants while participating in VIRDE. “*Mentoring during VIRDE”* and “*VIRDE lectures”* were the most selected responses (89.1, 78.3% respectively). “*Personal motivation”* (56.5%), “*protected work time during VIRDE”* (45.7%), “*interactions with VIRDE peers”* (39.1%), and “*network connections developed during VIRDE”* (32.6%) were also listed as enablers of their success.

### Knowledge in grant writing and research ethics

**Fig. 1:**
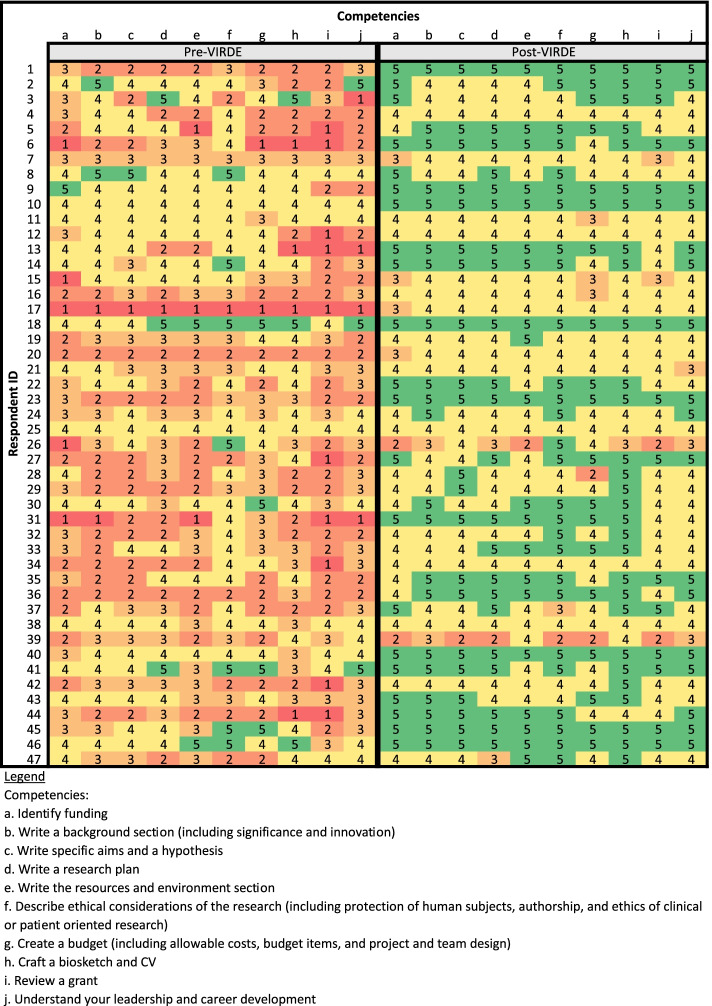
Heat Map of Grant Writing Knowledge Gained, Vanderbilt Institute for Research Development and Ethics (VIRDE)

Respondents reported on a scale of 1 (strongly disagree) to 5 (strongly agree) their grant writing knowledge across ten competencies pre- and post-VIRDE (Fig. 1). The respondents’ ratings of their post-VIRDE grant writing knowledge were significantly greater than the pre-VIRDE ratings for all grant writing knowledge questions (Paired t-test: *p* < 0.001). The average grant knowledge score pre-VIRDE was 3.0 and post-VIRDE was 4.4, an increase of 44%. The largest knowledge gains were in “*reviewing a grant”* (77.5% increase), “*creating a biosketch or CV”* (52.1%), and “*writing the resources and environment section”* (50.4%).

Respondents ranked their level of knowledge on ten grant writing competencies using a five-point Likert-scale (1/red = strongly disagree; 5/green = strongly agree). Paired sample t-test for composite comparison of pre/post test scores: *p* < 0.001.

**Fig. 2:**
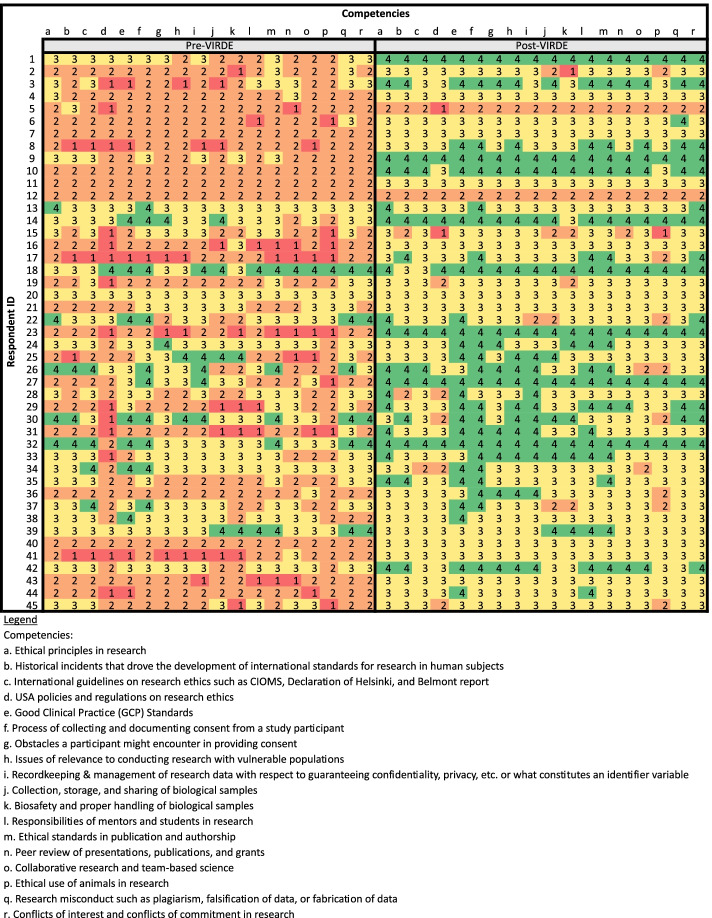
Heat Map of Ethics Knowledge Gained, Vanderbilt Institute for Research Development and Ethics (VIRDE)

Respondents also reported their pre- and post-VIRDE knowledge about research ethics across 18 competencies on a scale of 1 (uninformed) to 4 (expert) (Fig. 2). The respondents’ ratings of their post-VIRDE knowledge of research ethics were significantly greater than the pre-VIRDE ratings for all grant writing knowledge questions (Paired t-test: *p* < 0.001). The average research ethics score pre-VIRDE was 2.4 and post-VIRDE was 3.2, a 33% increase. In the pre-VIRDE section, there were 78 “*uninformed”* responses, compared to just 4 in the post-VIRDE section. The biggest changes were in “*US policies and regulations on research ethics”* (55.3% increase), “*responsibilities of mentors and students in research”* (44.8%), and “*Good Clinical Practice (GCP) Standards”* (41.8%). Additionally, 86.7% of participants indicated that VIRDE impacted their knowledge of these ethics topics “*a lot*.”

Respondents ranked their level of knowledge on 18 research ethics competencies using a four-point Likert-scale (1/red = uniformed; 4/green = expert). Paired sample t-test for composite comparison of pre/post test scores: *p* < 0.001.

### Sharing knowledge and collaboration

Respondents also reported increases in mentoring and teaching of others in their home institutions about grant writing. Only two scholars (4.3%) reported teaching courses about grant writing prior to VIRDE, and that number increased to 12 (25.5%) after VIRDE. Similarly, prior to VIRDE, eight respondents (17.0%) mentored others in grant writing, which increased to 31 (67.4%) post-VIRDE.

Before VIRDE, 36 respondents (76.6%) reported collaborating on research at organizational, regional, and/or international levels. After VIRDE, 46 (97.9%) respondents reported collaborating on at least one of these levels. The rate of collaboration increased by 27.3% at the organizational level, 40.0% at the regional level, and 44.8% at the international level.

### Grant productivity

Of the 47 respondents, only 15 (31.9%) had ever submitted a grant proposal for funding prior to their participation in VIRDE (Table 2). Pre-VIRDE, these 15 respondents submitted 53 proposals, of which 36 (67.9%) were awarded. However, in the period following participation in VIRDE, 39 (82.9%) respondents had submitted a total of 147 grants, of which 67 (45.6%) have been awarded at the time of the evaluation. Sixty-four percent of those who submitted at least one grant post-VIRDE were awarded at least one grant. The average grant award success rate per respondent post-VIRDE was 46.8%. The average time from the respondent’s participation in VIRDE to an awarded grant was roughly 2 years.

**Table 2:** Grant Productivity and Leadership, Vanderbilt Institute for Research Development and Ethics (VIRDE)

Indicator	Prior to VIRDE	After VIRDE
Individuals who submitted grants (*n* = 47)	15 (31.9%)	39 (82.9%)
Total grants submitted	53	147
Number of individuals by number of grants submitted		
*1 grant*	2 (13.3%)	6 (15.4%)
*2–5 grants*	7 (46.6%)	27 (69.2%)
*6–9 grants*	6 (40.0%)	3 (7.7%)
*10+ grants*	0 (0%)	3 (7.7%)
Individuals awarded grants	14	33
*Percent of those who submitted grants*	93.3% (*n* = 15)	84.6% (*n* = 39)
*Percent of the total respondents*	29.8% (n = 47)	70.2% (n = 47)
Total grants awarded	36 (67.9%)	67 (45.6%)
Number of individuals by number of grants awarded		
*1 grant*	4 (26.6%) (n = 15)	16 (41%) (n = 39)
*2–4 grants*	8 (53.3%) (n = 15)	15 (45.5%) (n = 39)
*5+ grants*	2 (13.3%) (n = 15)	2 (6.1%) (n = 39)
Role in grant (grants awarded)		
*Principal investigator (PI)*	22 (61.1%)	29 (43.3%)
*Co-PI*	10 (27.8%)	23 (40.3%)
*Multi-PI*	3 (8.3%)	6 (9.0%)
*Project manager*	1 (2.8%)	5 (7.5%)
*Other*	0 (0%)	4 (6.0%)
Funding mechanism (grants awarded)		
*United States of America government*	14 (38.9%)	27 (40.3%)
*European government or foundation*	11 (30.6%)	7 (10.4%)
*National organization*	4 (11.1%)	12 (17.9%)
*Foundation*	4 (11.1%)	9 (13.4%)
*Pharmaceutical company*	1 (2.8%)	5 (7.5%)
*Multilateral organization*	0 (0%)	5 (7.5%)
*Other*	2 (5.6%)	2 (3.0%)

We analyzed the types of funding agencies that supported the awarded grants. United States Government (USG) agencies supported around 40% of grants both before and after VIRDE. However, post-VIRDE there was increased diversity in other sources of funding, such as foundations and pharmaceutical companies, and about 18% came from national organizations, which was an increase over pre-VIRDE. Almost two-thirds (63.6%) of the respondents’ first awarded grant post-VIRDE was a one-to-two-year grant.

We evaluated demographic differences between respondents who did submit (39, 83.0%) and those that had not yet submitted (8, 17.0%) grants after VIRDE (Table 3). Sex was equally split among those that did not submit grants post-VIRDE, however, this distribution represents a higher proportion of females not submitting grants since only one-third of our respondents were female. Two of the eight alumni who had not yet submitted a grant proposal only recently participated in VIRDE in 2019. Three of the eight are employed in academia, three with a government institution, and two with an international non-government organization. Of those who did not submit grants, four hold a master’s degree as their highest degree, three hold a MBBS/MBChB degree, and one holds a bachelor’s degree. All respondents holding PhD degrees had submitted a grant proposal, as did 33 of the 37 (89.2%) respondents working in academia.

**Table 3:** Demographic differences between trainees who submitted grants and those who did not submit grants post-VIRDE

Category	Submitted grants (n, % of row total)	Did not submit grants (n, % of row total)
Total	39 (83.0%)	8 (17.0%)
*Female*	11 (73.3%)	4 (26.7%)
*Male*	28 (87.5%)	4 (12.5%)
Employment sector (respondents could check multiple sectors)		
*Academia*	32 (91.4%)	3 (8.6%)
*Government*	7 (70%)	3 (30%)
*Non-government (domestic)*	4 (100%)	0 (0%)
*Non-government (international)*	1 (33.3%)	2 (66.7%)
Highest degree attained		
*MD, MBBS, or equivalent*	16 (84.2%)	3 (15.8%)
*PhD*	17 (100%)	0 (0%)
*Master degree*	6 (60%)	4 (40%)
*Bachelor degree*	0 (0%)	1 (100%)

### Leadership roles and policy change

All respondents serve or served as a PI, Co-PI, or Multi-PI on at least one of their awarded grants. Most respondents (37, 77.1%) held leadership roles at their organizations. Among those working in academia, 13 (35.1%) respondents serve or served as a Department Chair, Dean, Deputy Dean of Research, or in another similar administrative leadership capacity. No respondents held solely a national or international leadership position, but nine (19%) held leadership roles at organizational, national, and international levels. More individuals reported that their research outputs impacted policy changes post-VIRDE (20, 42.6%) than pre-VIRDE (15, 31.9%).

### In their own words: the impact of VIRDE

#### Policy changes

Post-VIRDE, 20 respondents reported that their research contributed to policy change. In open-ended responses, they described changes made at three levels: clinic management and hospital; local and national; and regional and international. In clinic management and at the hospital, respondents helped develop new guidelines and protocols for the clinical management and treatment of diseases across a variety of medical specialties. At local and national levels, their research helped influence the adoption of new standards of care for sickle cell disease, change HIV treatment guidelines, advance a testing platform for syphilis during pregnancy, and provide free hydroxyurea for stroke prevention. Further, respondents helped improve tobacco control policies and establish a national non-communicable disease research database. Internationally, one respondent’s research helped change the World Health Organization (WHO) guidelines on the medical eligibility criteria for contraceptive use. Another respondent is helping to establish a digital surveillance platform for anti-microbial resistance in sub-Saharan Africa, while another wrote that “*results from our multi-center stroke prevention trial will change the narrative on and practice of stroke prevention in children with sickle cell disease in resource-constrained regions*.”

#### Career advancement

When asked about the impact that VIRDE had on their career advancement, open-ended responses (*n* = 43) were centralized around the skills and knowledge respondents gained that influenced their ability to write fundable grants. As one respondent explained, these improved research outputs “*enhanced my career and promotion prospects*” and other respondents were able to “*advance from lecturer to senior lecturer.*” These sentiments were referenced in similar ways by 13 respondents (30.2%). Seventeen respondents (39.5%) wrote about their success in receiving grants and 9 (20.9%) wrote about increased knowledge and skills to apply for grants and receive research funding. Through VIRDE, they “*have learnt new approaches to searching different funding sources, (and the) technical issues of NIH grants.”* Other respondents commented on awards they received to attend additional training programs and enrolment in doctoral programs because of their increased competencies from VIRDE.

Some respondents wrote about increased motivation, perseverance, and focus, with descriptions like “*VIRDE motivated me to make progress*,” “*I learnt how to persist despite difficulties*,” and “*I am more focused on my academic career*.” Respondents also noted ways that the training enhanced their teaching and outreach in the community, thus extending the impact of VIRDE. One respondent noted, “*I turned (into) a grant mobilizer and community leader in research and health program delivery.*”

## Discussion

### Findings

Based on evaluation data, VIRDE scholars develop skills and knowledge in grant writing and research ethics during this month-long immersive training program. While at VIRDE, they grow their professional networks and return to their home institutions where many successfully write and submit grants. For some scholars, this grant-funded research has resulted in changes to health care practice and policies. Alumni mentor and teach grantsmanship to colleagues, sustaining and promoting a research culture in their institutions.

Given the differences in research environments between LMICs and HICs, outcome measures commonly used in HICs, including number of publications in peer-reviewed journals, conference presentations, and funded grant applications, may not reflect accurately research capacity and productivity for scholars in LMICs. Cooke’s framework for research capacity strengthening provided an objective, non-traditional tool to assess the VIRDE program. Success of alumni and the program were broadly measured by scholars’ knowledge and skill enhancement, increased research collaborations, sustained engagement in research, and impact of research grants on health care practice and policies.

Gains in grant writing and research ethics knowledge as well as an increase in mentoring and teaching of these skills suggest that VIRDE scholars built skills and confidence in grant writing during the program. Respondents reported their largest gains in grant writing knowledge were related to reviewing a grant proposal and in the writing of specific sections of a grant proposal. Lectures and assignments throughout the one-month practicum focused on honing the scholars’ skills in developing each section of a grant application, culminating in a mock NIH-style grant review. Throughout the process of developing a grant proposal, scholars received frequent guidance from their mentoring committee. Reported gains in knowledge along with high ranking of VIRDE mentorship and lectures affirm the utility of hands-on, mentored practice combined with instruction.

We found a correlation between participation in VIRDE and increased research collaborations with international partner institutions. Partnerships are the foundation of the VIRDE program. VIRDE scholars are selected from institutions with existing collaborations with Vanderbilt University, as such it is not surprising that respondents reported being engaged in international collaborations. However, we found that post-VIRDE, the number of scholars engaged in international as well as institutional and national collaborations increased. The greatest increase in collaborations was at the international level, which could be attributed to VIRDE’s multidisciplinary and multi-country cohorts of scholars who work closely together during the month-long training, as well as scholars’ access to new mentors and exposure to research facilities at Vanderbilt during VIRDE.

The VIRDE program was founded upon a vision of training continuity and research sustainability. Survey results highlight the ripple effects of knowledge transfer as VIRDE alumni increased their engagement in teaching and mentoring in grant writing at their institutions. Sharing knowledge about grant writing has a two-fold impact. First, colleagues, mentees, and trainees learn about and improve their skills in grant writing, thus widening the impact of the program without additional program resources. Second, through teaching and mentoring, VIRDE scholars solidify their knowledge and establish themselves as leaders in grant writing. VIRDE alumni receive all training resources, including course slides. Providing course materials and continued technical assistance to alumni equips them to develop their own grantsmanship trainings adapted to their setting, which has been accomplished by some alumni.

Through increased grant funding, opportunities are created for alumni to both focus on and sustain their research careers. The data revealed that nearly three times more grants were submitted post-VIRDE, including 24 scholars who submitted their first grant proposal. In addition, post-VIRDE the diversity of funding organizations from which scholars applied to for grant funding increased, demonstrating new paths to sustainability and research independence for many individuals. Almost two-thirds of scholars who submitted grant proposals were awarded at least one grant and overall, half of submitted grants were funded. Respondents highlighted renewed focus, motivation, and perseverance in their pursuit of a research career as a result of participating in VIRDE. Such character growth can have a lasting positive impact on a scholar’s career trajectory. Incorporating mentoring and training to enhance these traits may contribute to scholars’ successes in grant writing, leadership, and research dissemination.

A substantial proportion of respondents have advanced in their career stage, serve in leadership roles, and continue to work in academia in their country of origin, contributing to a low rate of “brain drain.” Brain drain is common in LMICs as trainees often leave their country for career opportunities abroad. However, our data suggests that VIRDE equips scholars with skills, confidence, and networks needed to create the opportunities that lead to sustaining a research career while remaining in their home countries. Applicants are vetted first by an in-country team comprised of principal investigators who have existing partnerships with Vanderbilt before applying to VIRDE. This in-country process likely increases the probability of supported scholars who are connected to local networks to remain in academic research. Local collaboration for training is helpful to create sustainable, supportive research environments for scholars post-training.

Applicants are vetted for their ability to generate innovative research that is relevant to their setting. While traditional research dissemination metrics focus on publications in academic journals, we focus on the ability to sustain a research career through grant funding and for research to improve practice and health outcomes. Through evidence generated in their grant funded research, scholars reported changing practice, implementing policies, and impacting patients’ quality of life. Notably, scholars described that their research contributed to improving medical diagnostic and treatment protocols and other health-related policies in their regions, demonstrating the relevance and importance of their research. Scholars also contributed to international policy decision making groups, broadening the impact of their research to improve practice in other regions. These output metrics could be attributed to scholars’ increased grant productivity, collaborations, and confidence post-VIRDE, revealing the ability of this program to serve as a catalyst for scholars to develop research that is close to practice and responds to community and patient needs.

### Implications

We have highlighted elements beyond traditional metrics that indicate success of alumni and this program. These metrics, which include enhancing grant writing skills; expanding collaborations and roles as mentors, teachers, and leaders in grant writing; remaining in research; submitting grants; and impacting local and international practice, build upon Cooke’s framework and could be used by other programs to measure success. Key program elements that contributed to the program’s success include 1) a rigorous application and selection process led by LMIC partners requiring applicants to submit a draft grant proposal as part of the application process; 2) a curriculum based on applicable grant-writing skills taught by senior research faculty and staff combined with proposal development milestones; 3) committed in-country and U.S. based mentorship teams during and after VIRDE; 4) protected writing time away from competing work duties; and 5) an immersive cohort experience that provided participants with expanded professional networks.

VIRDE is a one-month program and grant development and submission can take months to years. Hence, based on the evaluation results, participating in VIRDE could be considered as a catalyst for success in grant writing. We acknowledge that VIRDE may not be the sole reason for their successes. Therefore, we queried scholars on their perception of VIRDE’s impact on their career and research productivity, as well as other factors that contributed to their success outside of VIRDE. Among the factors that enabled them to pursue research, at least half of respondents selected “*a personal desire to do research*,” “*a positive research culture at their institution*,” and “*mentorship*.” Success as an independent investigator also requires intrinsic motivation, determination, and perseverance, especially in a LMIC setting where respondents reported systemic challenges.

As we plan for the next 10 years, we are using this data to develop programming that continues to be relevant and effective. To date, only 31% of VIRDE participants were women. We recognize that VIRDE’s current format, requiring participants to spend one-month away from home, poses a challenge for gender equity among participants. Additionally, a one-month program in the U.S. can be costly and a prohibiting factor for researchers in LMICs. At present, VIRDE requires a funding source for participant scholarships as well as institutional support for program instructors and mentors. VIRDE scholars have predominantly been supported by NIH training grants, therefore limiting the number and breadth of participants. To address gender equity and cost, we are exploring incorporating online modules to decrease expense and duration of time in the U. S for those who cannot attend the entire month in-person. Further, for partner institutions which have a nucleus of VIRDE alumni and a more mature research portfolio, we are exploring transitioning some, or all, of the program elements to be taught in-country. We found that post-VIRDE, 25% of alumni taught grant-writing courses and 67% provided grant writing mentoring, demonstrating feasibility of expanding VIRDE through alumni trainers. With these cadres of research scholars trained in grant writing, VIRDE will continue to build individual grant writing skills among future participants while supporting alumni to develop and lead courses in the next phase of VIRDE. With networks of individual research scholars trained in grant writing, strengthening institutional research systems is the next step. We are working with alumni to develop programs at their institutions that strengthen indigenous grants management support [47].

### Limitations

We noted limitations in our data collection and analysis. Data related to knowledge and behavior were self-reported. Therefore, we relied on an accurate representation from respondents about their level of knowledge, participation in activities, and grant submission and awards. We also asked respondents to recall their knowledge and activities pre-VIRDE and differentiate that information from post-VIRDE knowledge and behavior. Participant recall on retrospective data may be biased. Additionally, respondents may have provided biased responses knowing that the survey came from the program director with whom many alumni are still in contact and that responses may be identifiable due to the relatively small sample size. Respondents may have wished to present their knowledge, skills, and outputs in a positive light. However, we believe that knowing the survey’s origins may have increased their willingness to participate in the survey, thus increasing the response rate. The survey included over 200 answer fields and survey fatigue may have affected responses towards the end of the survey. Since not all alumni responded, the actual program impact could be different. In reflecting on the survey design, respondents were not asked about challenges and areas for improvement associated with participating in the program, writing grants, or their research career. Such questions would have provided critical feedback and additional insight to VIRDE and grant writing for these researchers. Finally, these results are applicable to VIRDE scholars and findings may not be generalizable to the larger population.

## Conclusions

Our program evaluation data indicate that the VIRDE program contributed to increases in scholars’ knowledge in grant writing and research ethics. With increased knowledge, skills, and confidence in those areas, teaching and mentoring activities increased. After the program, scholars also increased their collaborations at local, national, and international levels and held more leadership roles. Fulfilling the aims of the program, scholars’ rates of grant submissions and awards increased, reflecting scholars’ ability to grow and sustain their research career. Scholars’ funded research also influenced community health outcomes and national and international health policies. Over the past decade, VIRDE has strengthened the research grant writing capacities of nearly six dozen biomedical investigators from LMICs, and has impacted positively on trainees’ health structures and research systems on many levels. Our experience can be used to inform the development, implementation, and long-term evaluation of similar research capacity training programs in under-resourced settings.

## Data Availability

The datasets used and/or analyzed during the current study are available by accessing the publicly available repository https://vumc.box.com/s/muztsnpjfsvn9ykn3r6hqarbwsdxp80a.

## Abbreviations

LMIC: Low- and middle-income country
HIC: High-income country
NIH: National Institutes of Health
VIRDE: Vanderbilt Institute in Research Development and Ethics
